# Self-Concern Across Scales: A Biologically Inspired Direction for Embodied Artificial Intelligence

**DOI:** 10.3389/fnbot.2022.857614

**Published:** 2022-04-25

**Authors:** Matthew Sims

**Affiliations:** Institute for Philosophy II, Ruhr-University Bochum, Bochum, Germany

**Keywords:** homeostasis, embodied cognition, anticipatory control, artificial intelligence, artificial symbioses, basal cognition, common fate, goal directed behaviour

## Abstract

Intelligence in current AI research is measured according to designer-assigned tasks that lack any relevance for an agent itself. As such, tasks and their evaluation reveal a lot more about our intelligence than the possible intelligence of agents that we design and evaluate. As a possible first step in remedying this, this article introduces the notion of “self-concern,” a property of a complex system that describes its tendency to bring about states that are compatible with its continued self-maintenance. Self-concern, as argued, is the foundation of the kind of basic intelligence found across all biological systems, because it reflects any such system's existential task of continued viability. This article aims to cautiously progress a few steps closer to a better understanding of some necessary organisational conditions that are central to self-concern in biological systems. By emulating these conditions in embodied AI, perhaps something like genuine self-concern can be implemented in machines, bringing AI one step closer to its original goal of emulating human-like intelligence.

## Introduction

Artificial intelligence (AI) was originally described as the project of making a machine behave in ways that would be called intelligent if a human were so behaving (McCarthy et al., 1955). Central to this notion of intelligence is the idea of task evaluation. True intelligent behaviour is read off from an agent's ability to successfully complete tasks requiring something akin to human cognitive capacities to be successfully completed.^1^ AI has generally fallen into two categories that align with two classes of tasks. The first category is *specialised AI*, which is designed with the aim of carrying out and being evaluated with respect to very *specific tasks* (e.g., playing GO, driving cars, generating language, etc.). *General AI*, on the other hand, is designed to carry out a broad domain of tasks that, at the time of design, are largely unknown (Thórisson et al., 2016). Although there is no agreed upon notion of intelligence in the AI literature, it is task evaluation, something that is often based on human psychological metrics that are used to determine whether a performance qualifies as intelligent or not. The Turing Test is a more general illustrative example of this manner of framing the concept of intelligence around the completion of tasks that are evaluated using human-based psychometrics (i.e., an artificial agent is intelligent if it can respond to a series of questions in a manner that is indistinguishable from responses of a human agent) (Turing, 1950)^1^.

Regardless of whether an AI agent has been designed to complete specialised tasks or even in the case of general AI, where tasks might not be fully known when designing the system, the general domain of tasks must eventually be recognised by designers to be evaluated. This suggests that tasks and their evaluation reveal a lot more about our intelligence than the possible intelligence of agents that we design and evaluate. Man and Damasio (2019), thus, ask the following important question regarding the legitimacy of using the completion of designer-based tasks as a reliable indicator of human-like intelligence:

“*Whose goals*?” Does an agent that myopically follows orders to the extent that it endangers itself and compromises its ability to carry out future orders deserve to be called intelligent?” (p. 447; author's emphasis).

An agent, for instance, that is equipped with a deep convolutional network and is able to correctly classify instances of threatening dogs when they are present 99% of the time in various environments but that fails in the capacity to behave in ways that allow it to avoid being damaged by a threatening dog also seems to lack something central to basic intelligence. Although such an agent might continue to complete the “visual” classification task that it has been given, the fact the completion of that task is compatible with the agent blindly pursuing its own destruction is at odds with the intuitive idea that basic intelligent behaviour is something that in living systems is typically adaptive and supports self-maintenance.

There is another approach to tasks and intelligence, however. This alternative approach suggests evaluating intelligence according to how a system completes tasks that bring about its own goals. It is becoming increasingly recognised that plants (Baluška and Mancuso, 2009; Shemesh et al., 2010; Trewavas, 2014; Gagliano et al., 2016; Novoplansky, 2016; Calvo et al., 2020) and basal systems (Maturana and Varela, 1980; Nakagaki et al., 2000; Hellingwerf, 2005; Ben Jacob et al., 2006; Lyon, 2006; Van Duijn et al., 2006; Shapiro, 2007; Saigusa et al., 2008; Baluška and Levin, 2016; Pinto and Mascher, 2016; Reid and Latty, 2016; Levin, 2019; Bechtel and Bich, 2021; Boussard et al., 2021; Hanson, 2021; Lyon et al., 2021) display some degree of intelligence that is expressed in various manners in which they adapt to the complexity of their environments. Selective pressures and environmental stresses that challenge both homeostasis and development are fundamental existential tasks that all biological systems encounter and must adaptively respond to. Importantly, remaining in a limited and select range of viability supporting physiological states is something that every biological system has a concern for (Jonas, 1966/2001). As such, any biological system is motivated from its genesis to deal with existential tasks that are intrinsic to it (Barrett, 2019). A system's own tasks are an expression of what Kant called “purposiveness without purpose” (1790/2007).^2^ All other tasks are organised around the task of continued self-maintenance, a task that endows both the environment and an organism's environmental interactions with meaning from its perspective.

The tendency of a system to bring about states that are compatible with its continued self-maintenance when perturbed is what I shall refer to as self-concern. Because self-concern is underwritten by a system's ability to measure and track the evolution of its own physical states and compare these with encoded optimal states, such concern may be said to reflect a form of self-reference. That said, self-concern should not be understood as involving the occasioning of personal-level states (e.g., beliefs and/or desires of folk-psychology) about the self or self-awareness. As it is being envisioned here, self-concern is strictly a subpersonal-level phenomenon, which can diverge from concern at the personal level (e.g., various systems that one's body is composed may slowly fail to track and return to states that are compatible with one's continued viability due to senescence; however, one during this period of development may be concerned for one's own survival). Importantly, if it is assumed that this kind of concern forms the basis of biological intelligence (something that will later be argued is, in fact, a reasonable assumption) and that biological intelligence offers a powerful and revealing lens with which to view intelligence across the board, then the following question becomes an emphatic one: how do we go about designing self-concerned AI agents in a way that sets the stage for basic intelligence to emerge in such agents?

The aim of this article is to investigate and bring to light some features of the relationship between intelligence and self-concern in biological systems in a manner that can be used to inform AI research. Self-concern in biological intelligent systems, it shall be argued, presupposes some form of embodiment. It is largely in part due to an environment's long-term effects (beneficial or adverse) on a system's body encountered through the interface of that body that its environment comes to have a meaning for that system, a meaning that is reflected in viability-sustaining behaviour driven by self-concern. Furthermore, various intelligent adaptive strategies that contribute to continued metabolic functioning and boundary regulation, it shall be argued, are tantamount to a series of solutions to a system's own intrinsic task (i.e., goal) of continued self-maintenance. It will be argued that until agents are able to exhibit something like self-concern on multiple scales of their embodiment, the kind of specialised and general tasks used to evaluate AI will continue to be overly theory-laden reflections of our own intelligence. Otherwise stated, until AI agents are concerned about their continued self-maintenance, their behaviour will continue to be exclusively guided by the processing of syntactical information that has meaning to us rather than semantic information that is grounded in an AI agent's own viability conditions (Kolchinsky and Wolpert, 2018). My aim is to cautiously progress a few steps closer to a better understanding of some organisational conditions that are central to self-concern in biological systems. By emulating these conditions (and most likely some others) in embodied AI, perhaps something like genuine self-concern in machines can emerge, bringing AI one step closer to its original goal.

The rest of the article is organised as follows: Section A Biologically Inspired Functional Approach to Intelligence provides a description of the biologically inspired functional approach to intelligence and then turns to the central notion of concern, showing how intelligence and concern are related; Section Three Necessary Features for Self-Concern in Biological Systems focuses on three necessary features of concern found in living systems: “system-environment energetic traffic,” the system-initiated process of energy accrual and exchange that allows an agent to defend its systemic boundaries and maintain its functional organisation *via* self-production; “dual information-carrying nature of interfacing bodily elements,” the property of an embodied system to harvest information simultaneously about the states of its external environment and its internal states; “hierarchically structured systems that share a degree of common fate,” the spatially and temporally nested organisation of systemic parts, the interaction of which is mutually supportive on different timescales; Section When Anticipatory Dynamics Answer to Self-Concern then looks at how anticipatory behaviour is related to concern and what this means for designing embodied AI systems in a manner that will get them closer to being self-concerned adaptive systems. I conclude with some brief remarks about some various design challenges and ethical issues that arise when considering self-concern in AI.

## A Biologically Inspired Functional Approach to Intelligence

One manner of restricting the scope of tasks (and task evaluation) in a way that respects the fact that intelligent behaviour is, at its core, an adaptive strategy is to deploy a biological functional approach to the notion of intelligence when designing AI. Such a biologically inspired approach describes what intelligence does for an agent as opposed to defining intelligence relative to the purposes and interests of the designer. For example, Sejnowski (2018), in describing intelligence, states that it is a general capacity that “evolved in many species to solve the problems they faced to survive in their environmental niches” (p. 263). Intelligence, from this perspective, is seen, first and foremost, as a solution to a (moving) set of environmental challenges that a system must adaptively respond to in order to remain alive^3^. To be sure, this functional approach does not claim that intelligence is limited to solving survival-related environmental problems; rather, it makes a more modest claim that intelligence is, fundamentally, a strategy for coping with environmental complexity (Godfrey-Smith, 1996; cf. Lyon, 2006).

Importantly, characterising intelligence in terms of its evolutionary function means recognising that it is something that can only be defined in relation to the kind of environment that an embodied agent must deal with in order to survive^4^. Being embodied and embedded in an environment is a precondition for cognition and anything that might be accurately deemed intelligent behaviour (Bateson, 1972; Clark, 1997; Pfeifer and Bongard, 2006; Pfeifer et al., 2007; Pezzulo et al., 2011; Lara et al., 2018). As such, differences in agent morphology and niche will be reflected in different forms of intelligent behaviour. For example, human intelligence, given the specifics of the human niche, will be different in form than, say, the intelligence that allows bees to successfully navigate their bee niches, pursuing opportunities for action, what Gibson (1966) called “affordances,” and avoiding potential harm-inducing situations that are specific to bee-like animals. In characterising biological intelligence relative to an agent's continued survival in its environmental niche, the notion of intelligence is rendered a relational property as opposed to a capacity that can be understood (or investigated) in the abstraction of what intelligence is a response to. In approaching intelligence by way of its biological function, we glean some insight (with a fair amount of speculation of course) into conditions under which various forms of intelligence have been evolutionarily selected for and, hence, why such forms of intelligence (human or non-human) are present today.

Crucially, by deploying a biologically inspired functional approach to intelligence, AI is not restricted to using human intelligent behaviour as a gold standard; although the aim of AI may be to design agents whose intelligent behaviour is human-like, a functional approach to intelligence suggests a different (yet not exclusively so) starting point to the investigation of intelligence. In taking a “biogenic approach” (Lyon, 2006) and, thus, recognising cognition's fundamental role as an evolved adaptive strategy, one first carries out a detailed investigation of the simplest instances of intelligent behaviour in basal biological systems and then works up to the more complex cases of intelligence in humans. Intelligence is, thus, viewed as something that reflects an evolutionary continuity among different forms of life along common phylogenetic branches and across different branches; this is a recognition that various forms of cognition may be evolutionarily convergent strategies that have arisen many times in different phyla much like vision or breathing has arisen multiple times, taking different forms. Human intelligence is, thus, not different in kind from that of simple organisms but different in form (cf. Darwin, 1871). Such a functional approach to intelligence provides a much-needed conceptual and methodological basis for throwing light on the fact that there is a “spectrum of intelligent behaviour found in nature that artificial systems can learn from” (cf. Webb and Scutt, 2000; Dupeyroux et al., 2017; Sejnowski, 2018, p. 267).

One apparent snag in deploying a functional approach to inform the development of AI is this: AI agents, even when taking the form of embodied agents, are typically fully abiotic agents. They are neither subject to heritable mutation nor does the notion of selective fitness apply to them; they are neither subject to senescence nor do suffer death. Of course, evolutionary algorithms might simulate such processes, but the chosen parameters that define the simulation and the chosen desiderata used to score fitness in such simulations are just that, chosen by designers (but see Bongard and Levin, 2021; Lehman and Stanley, 2011). One of the reasons that is embedded in an environment might be thought of as a precondition for intelligent behaviour is the fact that the presence of uncertainty that is inherent in natural environments has real existential consequences for systems situated in these environments. Fitness, which may be construed as an inverse cost function, “is a concept that only has meaning in the context of a concrete set of constraints, either from the environment or from the system being optimised” (Sejnowski, 2018, p. 267). Conceding to all of this is, however, consistent with holding the claim that basing the notion of intelligence on adaptation does not place any actual biological requirements on AI. It is not necessary that an agent undergoes or partakes in exact processes that are central to adaptation in living systems; rather, in order for machines to exhibit intelligence in any manner comparable to the simplest expressions of biological intelligence (e.g., archaea, bacteria, slime mould, yeasts, living cells, etc.), it may be the case that they will have to engage in some processes that are functionally similar to those that are characteristic of living systems. The presence of such biologically inspired processes, I shall argue, is the bedrock on which self-concern in machines might arise.^5^

### Self-Concern in Intelligent Systems

Returning to Man and Damasio's point above a central aspect of intelligent behaviour is that the goals of a behaving system can be said to be that system's own. Goals may be construed as monitorable physical states that a system's continued functioning depends on and to which a self-organising system tends to return after perturbation. In living systems, such goals may be construed as “homeostatic imperatives” (Man and Damasio, 2019), which a system's self-concern is defined with respect to; it is a system's goal of self-maintenance, which endows its actions and the environment with meaning from its perspective. Deploying concepts from cybernetic control theory (Wiener, 1948; Ashby, 1952; Conant and Ashby, 1970; Bateson, 1972), self-concern can be associated with a regulating system's tendency to return to an optimal set point range that is consistent with its continued functioning.^6^ It is the concern that an agent has for stabilisation of its physiological states within its viable set point range that motivates not only the simplest expressions of intelligent behaviour but also acts as the foundation on which the most complex forms of intelligent behaviour are grounded.^7^

Behavioural avenues of self-concern mirror the open-ended definition of evolution. There are myriad ways that such concern might be behaviourally expressed, and the update of options available to mitigate self-concern at any given time is the result of a system's continuous dynamic exchange with its environment. To put it differently, self-concern is the driver of flexible and evolving behavioural solutions to homeostatic challenges posed by hostile environments. As such, it reflects the inherent creativity that some have acknowledged to be central to cognition and life (Kant, 1790/2007; Goodwin, 1978, 1994).

Although various architectures implementing theories such as optimal control theory (Berridge and Robinson, 2003; Sterling, 2012), drive reduction theory (Hull, 1943; Konidaris and Barto, 2006), and homeostatic reinforcement learning (Sutton and Barto, 1998; Oudeyer and Kaplan, 2007; Keramati and Gutkin, 2014) have placed homeostatic maintenance state front and centre as a driver of intelligent behaviour in agents, self-concern fails to be directly addressed in current AI research.^8^ Part of this may be due to the fact that the relationship between self-concern and intelligent behaviour in biological systems is, itself, poorly understood. One of the aims of this article is to correct this; integral to the development of basic intelligence in AI, the kind that is ubiquitous in the living world, is understanding the details of self-concern in biological systems and designing agents that can engage in anticipatory homeostatic error correction fuelled by functionally similar machine self-concern. The next section offers a possible starting point for such a biologically inspired approach to embodied AI.

## Three Necessary Features for Self-Concern in Biological Systems

In what follows, I shall present three related features that I will argue are necessary (but not sufficient) for the emergence of self-concern in biological systems. These features are:

- Controlled system-environment energy traffic.
- Dual information-carrying nature of interfacing bodily elements.
- The common fate of hierarchically structured systems.

It is my hope that by making these features explicit, they may be instructive for the designing and improvement of already existing designs of biologically inspired agents, advancing the field of embodied AI one step closer towards the emergence of self-concern in artificial agents.

### Controlled System-Environment Energy Traffic

The first feature that I will argue is central to self-concern in biological systems, controlled system-environment energy traffic. The specific form that this traffic takes in biological systems occurs in the service of metabolism, which is “the set of life-sustaining chemical reactions within living cells” (Lane, 2016, p. 295). The two types of reaction characteristic of metabolism across all life forms are “anabolic reactions” (i.e., storing energy in the form of synthesised adenosine triphosphate, ATP) and “catabolic reactions” (i.e., breaking down of ATP into ADP + Pto release energy for work). It is through the acquisition of resources (e.g., nutrients) that living systems, *via* the combination of these reactions, are able to maintain the physiological processes which underwrite their ability to remain far from thermodynamic equilibrium with their environments, temporarily flouting the second law of thermodynamics. For biotic self-organising systems, remaining far from thermodynamic equilibrium means remaining alive (Nicolis and Prigogine, 1977; Friston, 2012, 2019; Demirel, 2014). A system, by harvesting resources from its local environment, can fuel the metabolic processes that allow it to (a) generate itself materially (e.g., protein synthesis) and (b) maintain its organisation in the face of environmental perturbation and despite continuous material turnover; these are the respective processes of “self-production” and “self-maintenance,” which form the basis of Maturana and Varela's (1987) notion of “autopoiesis” (see also Gánti, 2003, Chemoton model).

It is the sense in which a system both actively pursues resources and actively directs how these resources are used (i.e., how self-production and self-maintenance play out) that system-environment energy traffic is controlled (cf. Bechtel and Bich, 2021). Why might the process of controlled energic traffic be required for self-concern? Phenomenologist Hans Jonas provides a hint when he writes:

“In order to change matter, the living form must have matter at its disposal, and it finds it outside of itself, in the foreign ‘world.' Thereby life is turned outward and towards the world in a peculiar relatedness of dependence and possibility. It wants to go out to where its means of satisfaction lie: its self-concern, active in the acquisition of new matter, is essential openness for the encounter of outer being.” (Jonas, 1966/2001, p. 84).

The core idea that Jonas so eloquently expresses is that one of the directions of energy traffic that metabolism presupposes (taking in raw materials as a source of energy) places any living system in a relationship of need with its milieu; this need fundamentally arises from the fact that living systems are subject to constant material turnover that can only occur when that system acquires new resources. As such, this need implies a concern on the part of the behaving system for fulfilling its metabolic demands. Taking this into consideration, we may say that controlled system-environment energy traffic presupposes certain dependence on the environment on the part of the traffic controlling system. This dependence suggests that these systems that seek out sources of energy that they, in turn (*via* metabolism), use for work have a basic concern for their continued existence. Such is the primitive goal that every living system is concerned to satisfy, a goal that intelligent behaviour answers to and that provides the metabolising energy trafficking system with a basic perspective on the world (Lyon, 2006; Lyon et al., 2021). If this claim is in the right ballpark, then how may it be used to inform the development of AI?

From what has been said, we may glean this: if an agent altogether lacks the need for system-environment energy traffic that allows it to both maintain and produce itself to some degree, then that agent also lacks the capacity to exhibit basic concern. Such an agent fails to be autonomous (cf. Kauffman, 2000). Any behaviour that may be usefully ascribed to it, no matter how intelligent such an agent may be judged to be, fails to be its behaviour, because it does not stem from or, importantly, answer to its own concern. To be sure, both biological systems and machines require energy to do useful work in any capacity. However, and this difference is telling, biological systems both constantly monitor their energy levels across different spatial and temporal scales and actively behave in ways to fulfil their energetic needs. Such behaviour is not merely foraging for sources of usable energy but also generating the very materials and processes that allow for such energy foraging to continue to occur. Moreover, although to different degrees, each nested constituent part of a biological system, when all is going well, both contributes to and benefits from controlled energy traffic. This multi-scale concern reflects a basic “self-similarity” that is unique (at least for now) to the hierarchical organisation of biological systems (Bongard and Levin, 2021) (more will be said about this below). It is for this reason that an individual's biological parts (i.e., cell, organs, etc.), each of which takes part in its own energetic processing (i.e., monitoring and regulation), allow for distributed (decentralised) control at a very fundamental level^9^.

This brings us to the following question: could a machine ever truly engage in controlled system-environment energy traffic with its environment? Sure, but probably not in the same manner that an organism can. Metabolism occurs on the nanoscale where “there is spontaneous motion, but there is enough structure and the relations between forces are such that a lot can happen, by biassing tendencies in random walks” (Godfrey-Smith, 2016, p. 5). The rapid development of nanobots (Berger, 2016; Service, 2016; Linke et al., 2020) and engineered nanomaterials (Galetti et al., 2019) suggests that operation on the nanoscale itself, however, fails to present an uncrossable boundary to imitating energy traffic in machines (but see Nicholson, 2020).^10^ Furthermore, ongoing advancements in self-replicating robots (for a detailed review, see Moses and Chirikjian, 2020) may be a promising starting point for the development of a basic form of artificial self-production.^11^ It is a starting point because there is a notable difference between what might roughly be self-replication in machines and self-producing machines; whereas the former involves the use of supplied raw building materials to produce other machines that are copies of themselves, the latter involves the continuous generation (synthesis) of a system's own parts. To date, self-replicating robots are still limited to using supplied resources and cannot self-produce these materials from the ground up (Schranz et al., 2020); in other words, they lack the kind of “operational closure” that is unique to autonomous autopoietic systems (Maturana and Varela, 1980).^12^

One thing, however, that is key to keep in mind is that a system that is not subject to material degeneration (at least on short timescales) is a system that does not require (artificial) self-production. In other words, when an agent can both undergo substantial wear and tear due to its behaviour and environmental perturbations and monitor its own material degeneration, then harvesting energy from environmental resources takes on a particular value for the system; it is a manner of contributing to its own persistence across material turnover. Such turnover is something that is ultimately linked to the materiality of an embodied agent. For this reason, a soft robotic implementation may be invaluable in providing some implementational conditions for material turnover to occur and, hence, provide a need for artificial self-production. The production and development of microbial fuel cells (MFCs) in robots (Ieropoulos et al., 2005; Philamore et al., 2016) seem to be a promising manner of getting energetically autonomous embodied AI off the ground *via* biological metabolism (more on this below). MFCs provide systems that use them with conditions for material turnover and a need for self-production (at the level of microbes in the fuel cell).

Does it matter that “artificial metabolism” in machines will most likely be quite different from biological metabolism when it comes to being the fundamental of an agent's concern? I would like to suggest that such a question should not be addressed prior to the advent of artificial metabolism in agents. If it is telling of anything, to decide beforehand reveals nothing more than a deep commitment to the use of *a priori* intuitions, intuitions that may or may not be hostage to a deeply ingrained “biocentrism” when it comes to metabolism and/or self-concern (cf. Meincke, 2018).

Let us now turn to the second necessary feature of self-concern in biological systems, which, I will argue, is also a prerequisite for basic intelligence in embodied AI.

### Dual Information-Carrying Nature of Interfacing Bodily Elements

Even at the most basal level, biological self-concern relies on a system having multiple sources of feedback from its internal and external environments. It is only through the evaluation of such feedback that a system's behaviour may be directed in one way or another to return it to states that are compatible with its continued existence (i.e., its set-point values). Self-concern, although a feature of an entire system, is something that each component part of a biological system contributes to *via* the registration of information regarding its current state or condition and the state of the environment that it interacts with. Of particular importance are the states of components that causally interface the system with its external milieu.

From bacteria to humans, the presence of some form of the membrane that separates a living system from its external environment is ubiquitous. For example, *Escherichia coli* has a cell membrane that acts as a boundary between its cytoplasm and the external (terrestrial or fluid) medium, the dynamics of which the bacterium must behaviourally adapt to. The cell membrane and the flagellar motor machinery that it houses not only transmit external mechanical forces (tension, compression, and shear) to the internal components of the cell (Dufrêne and Persat, 2020), but multiple kinds of transmembrane receptor proteins and dedicated sensors also allow environmental feedback in the form of chemical gradients (Macnab and Koshland, 1972), light (Fraikina et al., 2015), and mechanical force (Dufrêne and Persat, 2020). Importantly, it is *via* the effects of environmental stimuli on the cell membrane and other environment interfacing components (e.g., flagella) that various internal biochemical cascades arise that contribute to the bacteria's ability to cope with their environmental dynamics. Were this interface to become non-responsive, the *E. coli*'s responses to its environment would in effect become random and ineffective, and thus the bacterium would soon cease to be. Like the simple *E. coli*, all living systems' membranes act as a conduit for proximal information about their environment. This proximal information is relevant to the notion of concern because its arising presupposes that some external force or stimulus has made a sensory contact with the organism already by way of inducing a change in the state of the membrane.

Of particular importance is the presence of mechano-stimulation, because it requires direct contact with the membrane or other bodily components that interface with the environment (e.g., hair, feathers, antennae, flagella, cilia, etc.). Proximal stimulus detection is not only informative about the states of the world but also the states of the system itself. If an abnormally high amount of mechanical stress is exerted upon the plasma membrane of a eukaryotic cell, it would result in membrane breach and that cell's likely death (Cooper and McNeil, 2015). Less dramatically, too much concentrated friction against a restricted area of the human epidermis (e.g., the skin on the tip of the finger) would result in damage to the epidermal membrane itself. Thus, there is a dual information-carrying nature that the interfacing bodily elements of biological systems have: they carry information about the conditions of the environment and the condition of the very system to which these interfacing elements belong. This dual information-carrying nature of interfacing bodily elements, I would like to argue, is a second necessary feature for self-concern in biological systems; self-concern requires a structure through which multiple sources of environmental feedback are integrated and causally related to the condition of the system.

This feature places some important constraints on the materiality of embodied agents, a constraint that can be read-off of direct observation on how such interfacing bodily elements are implemented in biological systems. One observation is that the kinds of membranes that separate a biological system from its environment are deformable; their structure is responsive to changes in applied external and internal forces (tension, friction, and stress). Deformation of a material substrate not only implies a change in environmental conditions (forces applied) but also change in the structural condition of the deformed membrane and the system that possesses such membrane.

Another observation is that the kinds of membranes and interface components found in livings systems often exhibit material elasticity (i.e., having low elastic modulus). This condition is tightly (yet contingently) related to the property of deformability; after structural deformation due to change in environmental conditions (e.g., brief exertion of compressive force), materials that things like cell membranes or epidermal membranes are made of allowing for a system to return to its original structure. In other words, the materials allow for recovery from deformation. This is relevant for any system that uses information about the environment to guide its behaviour because if such a system not only detects deformation but also how long it takes to elastically recover from deformation, that system can use the time difference between deformation and recovery to direct its behaviour and measure the states of its own responsiveness, something that may be informative about damage; whereas a short (or regular) recovery time suggests that the condition of the material itself is suited for optimal performance, a long (or irregular) recovery time suggests that the material has perhaps a defect or structurally damaged and unsuitable for optimal performance.

A third and last observation regarding the kinds of membranes and interface components that separate a biological system from its environment is that they exhibit local transmission of causal effects. This is just to say that affecting one concentrated area in a membrane or other interface component is likely to also affect adjacent areas. This property is, of course, related to that of being deformable and elastic. For instance, dropping a weight on a taught sheet of rubber stretched across a frame does not only deform the area of the sheet where the weight meets the rubber but also increases the tension on the areas surrounding weight. Similarly, exerting mechanical force on a biological membrane does not only deform the point of contact but also affects the adjacent areas. This property of local transmission is important for biological systems, because it allows for information about the environment and the self to be distributed locally, and, hence, contributes to the biological system's ability to use different sources of information to monitor itself (its own current bodily conditions). To be sure, the property of local transmission that accompanies both elasticity and deformability suggests that any neat separation of exteroceptive and interoceptive (and proprioceptive) information may be an artificial one (see Gibson, 1966 for a similar remark about exteroception and proprioception).^13^

These three material properties characteristic of dual information-carrying interfacing bodily elements imply something crucial about the structure of an embodied AI if its structure is to support the emergence of self-concern, namely, it is (partly) due to the fact that biological systems are composed of biotic structures made of materials that are largely deformable, elastic, and transmit local causal effects that such systems are subject to damage that occurs on the timescale of living systems. This timescale of damage can be contrasted to the timescale of damage that would be sustained by a structure composed of non-deformable, rigid materials. Many of our longest-standing architectural constructions (e.g., pyramids and the Colosseum of Rome) have been constructed of rigid materials such as limestone and cement, and our more recent architectural constructions add the strength of steel. The durability of these materials is related to their rigidity (e.g., their high tensile strength and compressive strength). The kind of damage that they sustain takes the form of long-timescale processes of metal corrosion, aggregate expansion, and calcium and lime leaching, to name a few. Because of the deformable, elastic, and local transmission properties of materials that largely make up living bodies, the damage that things like cell membranes sustain might take the form of post elastic limit micro-tears or enzymatic decomposition (e.g., being digested by an amoeba). Since such damage is detectable and occurs at fast timescales (i.e., it is not due to being exposed to constant environmental conditions) it allows living systems to behave in ways that minimise future damage, fleeing the situation if motile, or nutating towards better conditions if sessile.^14^

Material properties that underwrite the ability of interfacing bodily elements to carry information about the world and the system itself are largely captured with new technologies of the rapidly developing field of soft robotics (Hawkes et al., 2017; Booth et al., 2018; Shih et al., 2019, 2020; Thuruthel et al., 2021; Hardman et al., 2022). Whereas traditional robotics, focusing upon task precision and strength in controlled environments, have used (and still use!) rigid metal links (i.e., joints) and electric non-distributed actuators, soft robotics focuses on the adaptability of robots to the real world and complex environments, and, as such, uses synthetic compliant materials that can undergo deformation along with distributed actuators. In addition to this, conductive piezoresistive strain sensor fibres have recently been incorporated into the self-healing deformable material, allowing for the control system to sense damage (Georgopoulou et al., 2021) and proprioceptive feedback (Truby et al., 2020).^15^ This feature seems to accurately map onto the material properties (deformability, elasticity, and local transmission of causal effects), which, as I have argued, are required for implementing interfacing bodily elements that carry information about both the environment and the agent. This being said, were such soft robots able to measure their own energetic conditions and actively (or proactively) engage in energy-sourcing and material exchange—the kind which we have already seen is suggested by controlled system-environment energy traffic, such robots would indeed be one step closer to the realisation of agents with self-concern (cf. Man and Damasio, 2019).

Let us now move on to the notion of a hierarchically structured organisation; the third and last feature that I will argue is necessary for concern in biological systems.

### Common Fate of Hierarchically Structured Systems

Living systems are self-organising complex adaptive systems. This means that they can maintain themselves in states that are far from thermodynamic equilibrium, temporarily avoiding the dissipation that inevitably results from the tendency for entropy to increase (Nicolis and Prigogine, 1977; Friston, 2012, 2019; Demirel, 2014). One central property of complex systems is that they are hierarchically organised (Simon, 1962). We may understand the notion of a hierarchical system in terms of “containment” where a larger system contains smaller subsystems nested within it (McShea, 2012). This is to say that such systems contain “interrelated subsystems, each of the latter being, in turn, hierarchic in structure until we reach some lowest level of elementary system” (Simon, 1962, p. 468).^16^ Multicellular organisms, for example, contain living organs and tissues that, in turn, contain living cells. Although the hierarchical organisation of complex systems more generally may be construed in terms of intensity of interactions (i.e., who interacts with whom and how often), the form that the intensity of interaction takes in the hierarchical organisation of both biological and physical systems is that of “relative spatial propinquity” (Simon, 1962, p. 469).

Hierarchical organisation implies differences in both relative timescales and behavioural constraints; faster timescale behaviour of nested subsystems is constrained by slower timescale behaviour of systems in which they are nested. Roughly, system Y is constrained by another system, X, just in case the latter's features act as order parameters for the former, reducing the degrees of freedom of Y.^17^ As such, we may say that the faster timescale dynamics of some nested system is “enslaved” to the slower global dynamics of the nesting system, making the former subordinate to the latter (Haken, 1985). For instance, the homeostatic condition of a liver (roughly, a collection of differentiated cells and biochemical cell interactions) may largely constrain the homeostatic state of any individual cell contained in the collection, and, simultaneously, the individual cells contribute to the maintenance of the liver that constrains them. Crucially, the kind of constraint relations that are governed by a biological system's homeostatic imperatives, unlike those in systems that do not answer to system-wide self-maintenance, means that “certain differences in the part have an informational effect upon the larger unit, and vice versa” (Bateson, 1972, p. 324). This is just to say that for both nested and nesting biological systems, a cause for homeostatic compensation at either of their respective levels is semantic information (Kolchinsky and Wolpert, 2018).

How is hierarchical organisation related to self-concern? To answer this question, let us first consider the fact that in multicellular biological systems every nested subordinate (bounded) system within a larger superordinate system is, itself, adaptive and “participates in its own self-maintenance, sensing and signalling the state of its life process” (Man and Damasio, 2019, p. 447). This suggests that self-concern is not merely a property of a superordinate system, but that it is something that arises at each nested level of biological organisation to varying degrees. Just as organisms adaptively respond to external environmental challenges (e.g., stresses) they face to avoid dyshomeostasis, organs and tissues modify their dynamics in ways that are adaptive to the challenging conditions of their body environment; similarly, bodily cells *via* variable gene expression biochemically respond to the challenges of their organ or tissue environments (Pezzulo and Levin, 2016; Levin, 2019).^18^

What about prokaryotic organisms that do not have organelles and yet still exhibit self-concern? Although elements within prokaryotes (e.g., microtubules and actin filaments of the cytoskeleton, etc.) may not exhibit self-concern themselves, something that follows from the fact that such parts fail to satisfy two necessary conditions for the self-concern proposed above, it is the dynamics occurring across different scales constrained by the temporal-spatial organisation of such elements that contribute to self-concern at the level of the organism. Importantly, these element dynamics may be characterised as serving a common fate; it is by contributing to the continued homeostatic maintenance of the whole organism that each element brings about the continued production of itself *via* the material turnaround that is insured by the continued functioning of the organism.

With these considerations in mind, I would like to suggest that self-concern is a system-level property that rests on there being a common fate of both the hierarchically nested parts and the nesting (i.e., constraining) system.^19^ Without some degree of common fate being respected at relatively lower levels of hierarchical organisation, self-concern at higher levels is not possible. Imagine the rather brutal example that tomorrow every cell in your body would begin competing for resources with one another; this would be tantamount to each cell behaving as a unicellular organism (ignoring the presence of biofilms for the sake of argument!). Although each of these cells might exhibit self-concern in much the same manner that prokaryotes do, self-concern would fail to arise at the level of either the organ or the organism. Each cell would fail to contribute to the self-maintenance of your organs (or you at the organismic level) because of the absence of common cell fate; the viability of one cell in such a brutal case may indeed be largely dependent on bringing about conditions that would adversely affect the homeostasis of all others. Thus, self-concern in biological systems at the level of nesting (constraining) system requires that nested systems share some degree of common fate with nesting systems.

If the common fate of hierarchically organised systems is a requirement on biological self-concern, and such concern is a requirement on biological intelligent behaviour, then using the biological case as a model for agents, it seems that some degree of hierarchically organised systems exhibiting common fate will be a necessary feature for embodied AI if something even slightly akin to basic biological intelligence can be exhibited by AI. How can the notion of the common fate of hierarchically organised systems inform embodied AI more specifically?^20^ One very general suggestion is to explore the endosymbiotic relationship and design artificial endosymbiotic systems. The idea is to somehow elicit self-concern at the level of global nesting-system from both (a) the interactivity of the global system and a simple nested system, which have both been programmed to emulate a form of mutualism (i.e., to use and contribute to one another's continued self-maintenance) and (b) the agent-environment energy (metabolic) traffic that the global system is forced to engage in to keep the endosymbiont and itself functioning. Treating the endosymbiont agent as the lower, faster scale subordinate system and the host agent as the slower scale superordinate system, the hierarchical organisation of these systems sharing a degree of common fate, amongst some of the other features that we have already touched upon, may support a self-organising autonomous system, a system that by bootstrapping^21^ can cast away its scaffolding, replacing the self-maintenance tasks extrinsically put in place by designers with tasks that arise (i.e., are discovered) from interactions between the parts, tasks that reflect an intrinsic concern on the part of the whole system for its own self-maintenance.

Recent developments in swarm robotics may prove to be a promising avenue for testing this hypothesis (see Christensen et al., 2008; Liu and Winfield, 2009 for symbiotic-inspired robot swarms). Here, interaction among robots has taken the form of continuous (Bezzo et al., 2014) or pulse-coupled oscillatory signals (Barcis et al., 2019), whose alignment of oscillatory frequency and/or phase alignment allow members of a swarm to behave synchronously (Schranz et al., 2020). If swarm robots were to implement the kind of hierarchical common fate dynamics, which, as I have argued, is required for self-concern, then the interaction among them must not only take the form of information-sharing but must also involve behavioural interaction by exerting reciprocal mechanical forces that have consequences on the continued functioning of both the nested and nesting parts.

Another research area that may lend itself to the development of agents with common fate at various hierarchical levels of an organisation is that of hybrid associations composed of both machines and microorganisms (Ieropoulos et al., 2005; Philamore et al., 2016; Tsompanas et al., 2021). For example, anaerobic *anodophile* bacteria, when used in a microbial fuel cell (MFC), will transfer electrons to the MFC's electron-accepting anode electrode, which in turn supplies an MFC housing robot with electrical energy (Habermann and Pommer, 1991; Ieropolous et al., 2004).^22^ If such an MFC housing robot behaves so as to remain in (or return to) environments that are rich in kinds of substances that MFC-inhabiting bacteria can metabolise (e.g., sulphate, acetate, glucose), then its doing so mutually supports the survival of the bacteria and its own continued energetic functioning. Further development of this kind of (biotic/abiotic) artificial symbiosis between nested bacteria and a nesting machine, if what has been argued here is correct, may be one crucial method of bringing about self-concern in AI, namely, bacteria's self-concern may be the source of the emergence of self-concern in the larger hierarchically organised bacteria-machine system.

One important takeaway from this section is this: the fact that self-concern has not yet been implemented machines (Man and Damasio, 2019, p. 447) does not suggest that such self-concern cannot find expression in machines or organism-machine associations. If self-concern is a requirement for basic intelligence across the board, then we should expect a concentrated effort on the part of future research to develop hierarchically organised systems that implement self-concern across scales.

## When Anticipatory Dynamics Answer to Self-Concern

With three necessary features of biological self-concern on hand, I now want to consider the relationship between self-concern and intelligence by way of a particular capacity that is central to cognition: anticipatory behaviour. My argument in this final section will take the following form: anticipatory behaviour is often recognised as indicative of intelligence; an important aspect of intelligent anticipatory behaviour (in contrast to mere anticipatory behaviour) is that it is possible for such behaviour to answer to a system's long-term homeostasis; a system's behaviour that answers to its long-term homeostatic stability is behaviour that answers to that system's self-concern; therefore, an important aspect of intelligent anticipatory behaviour is that it is possible for such behaviour to answer to self-concern. If one effective way of bringing embodied AI closer to emulating the intelligence found in even the most basal of living systems is to use aspects of biological intelligence to inform AI design, then designing agents, the anticipatory behaviour of which could answer to self-concern, may be one manner of bringing AI closer to emulating the kind of intelligence observed in the biological world.

It has been widely acknowledged that one way of expressing intelligence in biological systems is by an exhibition of anticipatory behaviour (Bartlett, 1932; Craik, 1943; Piaget, 1970; Neisser, 1976; Drescher, 1991; Arbib, 1992; Riegler, 2001; Grush, 2004; Castelfranchi, 2005; Lyon, 2006; Bar, 2007; Pezzulo, 2008; Bickhard, 2016; Nasuto and Hayashi, 2016; Levin, 2019; Kiverstein and Sims, 2021; Sims, 2021). Anticipatory behaviour may be generally characterised as a behaviour that allows for a system to respond to yet-to-be encountered changes in external or internal environmental states as a function of prior states that the system has encountered. This characterisation throws light on one reason why anticipatory behaviour is considered as an expression of intelligence: it involves deploying some form of memory and learning (or model acquisition more generally) to bias behaviour towards a system-preferred outcome; hence, it involves some form of information processing. Such behaviour is orchestrated in a manner that is dependent on internal states that have a certain degree of independence or detachment from current streams of sensory information (Pezzulo, 2008). Internal states may be generally construed as constituting a system's “internal model” that captures environmental dynamics and the effects of its actions on its environment (Neisser, 1976; Rosen, 1985/2012; Pezzulo, 2008; Friston, 2012; Pezzulo and Levin, 2016; Schulkin and Sterling, 2019).

Anticipatory behaviour, when all goes well, delivers preferred behavioural outcomes. Such outcomes are relative to physiological states that a system should visit given both its phenotype and the form of metabolic redox machinery that its phenotype serves (i.e., kinds of donors and acceptors a system implements to fuel proton chain reactions to drive catabolism of ATP). A preferred behavioural outcome for *E*. *coli*, which metabolises glucose, is encountering high concentrations of glucose in its environment. On the other hand, a preferred behavioural outcome for a sun-loving plant such as *Portulaca oleracea*, which requires photosynthetic light to effectively convert H_2_O and CO_2_ into sugars, is encountering the presence of photosynthetic light. It is because preferences exhibit a high degree of stability and can act as reference points for long-term homeostatic supporting behaviour in the face of environmental flux (i.e., set-point values) that preferences themselves can be understood as (partly) constitutive of a system's internal model. Recall that long-term homeostasis is an intrinsic existential goal in all biological systems; as such, a system's behaviour that answers to its long-term homeostasis is a behaviour that answers to that system's self-concern (Jonas, 1966/2001). It is, thus, the ability for anticipatory behaviour to be driven by a system's concern for its own continued stability that qualifies such behaviour as intelligent at its most fundamental level. For example, a hypothetical system that only behaves anticipatorily, bringing about outcomes that are irrelevant to its continued survival, would certainly fail to survive very long; this would be because of the fact that no behaviour comes without some metabolic cost, and that regularly engaging in anticipatory behaviour that brings about expected sensory or behavioural outcomes is consistent with regularly bringing about maladaptive conditions. The regular and repeated proactive jumping of a mouse to the exact next location where a snake predator will strike falls short on any account of being an example of intelligent behaviour. Such a mouse, despite the accuracy of its predictions, is a dead mouse.

There is an objection waiting in the wings, which may be posed as follows: certainly, an elaborately planned suicide can be an exhibition of intelligence, and such a plan neither answers to long-term homeostasis nor self-concern on the part of the organism! This counterexample can only go through, however, if the claim was being made that all intelligent anticipatory behaviour must answer to a system's self-concern. The claim that I am making, however, is only that in order for some anticipatory behaviour in a biological system to qualify as intelligent, it must be possible for such behaviour to answer to self-concern grounded in the continued long-term homeostasis of that system.^23^ In other words, if a system's anticipatory behaviour could not, in principle, be influenced by its homeostatic norms, then such behaviour, although it is anticipatory, fails to exhibit the form of intelligence that is typical of biological anticipatory behaviour. Hence, even though an elaborately planned suicide results in loss of life and dyshomeostasis, it is the type of behaviour that could, in fact, answer to the maintenance of long-term homeostatic stability. On such an occasion, however, it simply fails to do so.

There have been a number of recent cognitive theories and computational models that have taken into account the role of anticipation for intelligent behaviour and that have been used to inform cognitive robotic technologies (see Nasuto and Hayashi, 2016 for an overview).^24^One increasingly popular process theory that has been used to formulate specific anticipation-driven cognitive architectures is active inference (Friston et al., 2009; Pezzulo et al., 2015; Morville et al., 2018; Baltieri and Buckley, 2019; Corcoran et al., 2020; Millidge, 2020). This theory, which was originally applied to brain dynamics, casts perception, action, and learning in terms of a Bayesian inference problem-and-error correction. In active inference, agents are endowed with prior beliefs that they will frequent some sensory states more than others, and these priors reflect an agent's homeostatic range. Unlike rewards in classical reinforcement learning, which are received from the environment, priors in active inference agents are internal states of the agent that can remain stable across environments and, hence, in this sense may be construed as intrinsic to the agent (Friston et al., 2015). Similarly, Keramati and Gutkin's (2014) homeostatic reinforcement learning model aims to make sense of how rewarding behavioural outcome values are computed as a function of internal states and estimate dyshomeostasis reduction in an outcome.

Intelligent anticipatory behaviour in AI requires that it is possible for behaviour to answer to an agent's own intrinsic homeostatic goals; such behaviour is grounded in self-concern. Whether or not anticipatory architectures such as active inference (or homeostatic reinforcement learning for that matter) will bring embodied AI closer to intrinsically emerging normativity is dependent on the ability of such architectures to provide agents with means to go beyond the values that designers initially endow them with. In the case of active inference, this will likely include providing agents with a means for discovering conjugate priors (i.e., hyperpriors) (see Sajid et al., 2021) within real world environments that pose concrete threats to the continued self-maintenance of the agent. This kind of adaptive plastic reshaping of priors (i.e., value) may be roughly conceptualised as a form of accelerated evolution where a single agent is seen as an evolving lineage subject to open-ended learning (Standish, 2003; Stanley et al., 2017). By equipping an agent with the necessary means to learn the normative parameters that define its continued self-maintenance in real-world environments, the anticipatory behaviour of such an embodied agent is poised to answer to its own self-concern. What more is required? If what I have presented above is accurate, then focusing on the further development of (1) controlled system-environment energy traffic, (2) dual information-carrying nature of interfacing bodily elements, and (3) highly integrated, hierarchically structured systems sharing a common fate will be necessary to bring the current AI, the various architectures of which are already capable of generating accurate estimations of yet-to-be encountered states, much closer to emulating the kind of intelligent behaviour exhibited by biological systems, a behaviour that is grounded in self-concern.

## Conclusion

In this article, I have argued for the centrality of concern in biological systems even for the most basal expressions of intelligent behaviour. I argued that if this is the case, then there is good reason to think that if the intelligence of an agent is to ever be comparable to the intelligence of a biological system (human or otherwise), then it will require the presence of some form of self-concern. I have described three necessary features underpinning the concern in biological systems that can be used to inform the development of functionally similar features in embodied AI. Lastly, I have argued that although anticipation may be seen as central to intelligent behaviour, it is only anticipatory behaviour that could answer to the intrinsic norms of the system and, thus, be subject to self-concern which is a clear exhibition of intelligence.

To close, let us look at a few questions that highlight some of the complex issues that arise when considering the possible implementation of self-concern in embodied AI. Although addressing these questions falls beyond the scope of this article, I would like to stress the importance of considering them.

Let us assume that we have managed to implement self-concern in an embodied AI. This agent will be very unlike any of those that we currently interact with in at least one central manner; a self-concerned embodied AI agent will do what it can to remain in states that are consistent with its continued functioning/existence. This hypothetical case provides us with the opportunity, in closing, to raise a few important questions on potential challenges and ethical issues regarding self-concerned AI.

One challenge is to design self-concerned embodied AI agents in a way that avoids deceiving us (a form of explanatory opacity *via* transmission of misinformation). This will be crucial for such AI agents given the fact that if they exhibit the kind of basic biological intelligence that I have argued arises with self-concern, such agents may, in fact, disguise their immediate goals (deception) in order to satisfy their long-term goal of continued self-maintenance, not unlike many living organisms do (e.g., mimicry, feigning death, etc.). A further challenge is this: one can easily imagine a case in which such a self-concerned AI agent's continued functioning might be incompatible (or evolves over time to be incompatible) with the continued well-being of a human (or multiple humans) (e.g., competition for common resources, etc.). How can this scenario be avoided *via* precautionary design efforts without jeopardising the very aim of self-concern in embodied AI? A related ethical question arises when considering that an embodied AI agent that implements self-concern need not be one that is self-aware or conscious. Should the fact that we are aware that such an AI agent has an intrinsic concern about its own continued functioning be enough for us to be obligated to avoid impeding its intrinsic goals? If so, what are the situations in which we can renege on such obligations?

One important question that we are left with is whether self-concern in embodied AI is something that could even be recognisable by us? Although it might take the form of machines that flexibly and adaptively behave in ways that allow them to avoid their own “machine death,” the kind of behaviour that will be driven by machine self-concern is likely to be very different from that which we can readily identify in living systems. Perhaps most interesting here is the prospect of learning more about the aspects of self-concern that are particular to humans from our attempts at making sense of self-concern in machines. Whatever the case, although the emergence of concern in AI may very well depend on us, once up and running, the intelligence that such systems exhibit will be directed at completing tasks that are (largely) their own.

## Author Contributions

The author confirms being the sole contributor of this work and has approved it for publication.

## Funding

This research has been supported by the Alexander Von Humboldt Foundation.

## Conflict of Interest

The author declares that the research was conducted in the absence of any commercial or financial relationships that could be construed as a potential conflict of interest.

## Publisher's Note

All claims expressed in this article are solely those of the authors and do not necessarily represent those of their affiliated organizations, or those of the publisher, the editors and the reviewers. Any product that may be evaluated in this article, or claim that may be made by its manufacturer, is not guaranteed or endorsed by the publisher.

## Footnotes

^1^Although “intelligence” is often used to refer to higher cognitive capacities in the literature, in what follows, the terms “intelligence” and “cognition” will be used synonymously.

^2^This is the idea that the worth of a purposive system resides in its own being rather than a result, the value of which is determined external to that system (Cassirer, 1981).

^3^Locating the notion of human intelligence in various abilities to cope with survival-relevant human environmental tasks, the aim of AI, as it was introduced by McCarthy et al. (1955), might be reframed as the project of getting machines to cope with human niches in ways that would be recognized as being analogous to how humans adapt to the complexity of our niches.

^4^The famous cyberneticist Bateson (1972), in many ways, auguring the arrival of embodied and situated cognition, recognized the importance of considering bodies and environments as parts of cognitive explanation. According to him, the cognitive system is a unit of explanation, the bounds of which are determined by information-carrying pathways (i.e., circuits) that cannot be severed without rendering the explanandum mysterious.

^5^This is particularly telling: if something like adaptivity is assumed to be a fundamental feature of all intelligent behaviours, then the progress of AI will not proceed the progress of artificial life (AL) or vice versa; the progress of both the AL and AI programmes will depend on their marching in lockstep.

^6^The notion of stable set points borrowed from the cybernetic control theory is a useful abstraction in the sense that homeostatic equilibria are not stable but shift across the lifetime of a biological system. Hence, homeostatic set points are more accurately seen as dynamic or moving equilibria.

^7^The fact that homeostasis is emphasized in this article as a basis for self-concern, however, does not imply that the process of heterostasis (i.e., exploring and seeking high information gain *via* self-perturbation) is not a significant driver of behaviour in self-concerned living systems.

^8^Oudeyer and Kaplan (2007) focus on the development of models of “intrinsic motivation” within the framework of reinforcement learning, and there may be some general overlap with what I am calling self-concern.

^9^One dimension in which system-environment energy traffic might vary in degree is with respect to how much each constituent part of a system both contributes to and relies on the energy traffic of the system (s) in which it is nested or coupled to. For example, if system-environment energy traffic is limited to only one nested element of a larger nesting system, then that larger system might satisfy the requirement of system-environment energy traffic but only to a minimal degree, whereas if each constituent and nested element of a larger system contributes to and relies on the energy traffic of all other elements (to varying degrees), the supraordinate system has a high degree of system-environment energy traffic. Quantifying this dimension of system-environment energy traffic falls out of the scope of this paper.

^10^Nanobots, however, are programmed to do specific tasks and, as such, they differ, at least currently, from, say, autonomous protein motors in biological systems (Linke et al., 2020).

^11^The theoretical beginnings of the current research programme of self-replicating machines may be traced back to Von Neumann's (1966) logical models of self-reproducing automata.

^12^Xenobots (Kriegman et al., 2020), algorithmically designed collections of frog skin and heart cells that have the capacity to heal themselves, may be a possible exception to this.

^13^Although I have focused on membranes and their properties that are typical to living systems that exhibit dual information carrying, having a membrane is not a necessary requirement for dual information carrying to be physically instantiated. As long as there are distributed sensing/actuation devices that can harness and leverage spatially relative information, dual information carrying can be instantiated by a network of elements that are not, as a whole, enveloped by a single membrane. In such cases, the boundary that is instantiated is one of dynamically coupled causal influence. For example, a swarm of robots need not itself have a membrane to exhibit dual information carrying. All that is required is that the behaviour of each component element of the swarm is a function of the sensing/actuation parameters of the others. In such a case, the coupled swarm will exhibit dual information carrying across its network of distributed swarm members, each with their own sensing/actuation devices that contribute to an emerging dynamically bounded system.

^14^This is not to suggest that living systems are not subject to slow and gradual decomposing *via* oxidation reactions; they are if they are aerobic respiring systems or they live in oxygen-rich environments. Such damage, however, being the result of a constant environmental condition, fails to be something that an organism can escape from; it is merely a condition on aerobic life. As such, although complex organisms like us are aware of the slow damage that oxidation causes, it is not something that we are concerned with given it is a condition on our aerobic life.

^15^The “Octobot” (Wehner et al., 2016) is a striking example of an entirely soft robot that contains no electronics. Much like signal processing in slime mould, the signal processing in the Octobot occurs *via* oscillations and fluid transport (microfluidic logic). Locomotion, signal processing, and decomposition of onboard fuel supply are closely connected!

^16^Of course, the identification of a lowest level is something that is, itself, an open question of scientific investigation and a matter of contention. However, for the purposes of this article, we can set the question of what such a level might be aside.

^17^In synergetics (Haken, 1985), an “order parameter” refers to a measure of a global system's slow macroscale dynamics that determines the fast dynamics of microscale component systems by reduction of the degrees of freedom of the latter.

^18^Parisi and Petrosino (2010) were early to stress how robotics could be instrumental for understanding how organisms adapt to both “external worlds” and “internal worlds.” Conversely but in a similar spirit, I am suggesting that understanding how hierarchically nested/nesting biological systems adapt simultaneously to both internal and external worlds is crucial for the development of concern in biologically inspired robots.

^19^The notion of common fate was originally introduced by Wilson and Sober (1989) in the context of providing a characterisation of biological individuals.

^20^It should be emphasised that the kind of nested hierarchical organisation of elements with common fate that I am arguing about is fundamental to (self) concerned systems involved in concrete physical implementation rather than merely a hierarchically organised network (architecture) that plays the role of a control system. Many thanks to Christian Oettmeier for pushing me to clarify this point.

^21^The term “bootstrapping” is used in a general manner here to denote a “process that automatically increases in complexity” (Moses and Chirikjian, 2020, p. 9) rather than the specific notion of bootstrapping deployed in statistics.

^22^MFCs are transducers that are given a biochemical energy source and convert it to electrical energy; they can, thus, power robots in which they are located.

^23^I am concerned here with nomological possibility and not logical possibility.

^24^The implementation of anticipatory dynamics, of course, is not new. In optimal control theory, forward models (i.e., internal models that generate predictions of the sensory consequences of motor commands) have been used at least since Jordan and Rumelhart (1992).
